# Weight loss and bone mineral density in obese adults: a longitudinal analysis of the influence of very low energy diets

**DOI:** 10.1186/s40842-018-0063-6

**Published:** 2018-06-19

**Authors:** Palak Choksi, Amy Rothberg, Andrew Kraftson, Nicole Miller, Katherine Zurales, Charles Burant, Catherine Van Poznak, Mark Peterson

**Affiliations:** 1 Department of Internal Medicine, University of Michigan, 24 Frank Lloyd Wright Dr, Ann Arbor, MI 48106 USA; 2 Molecular and Integrative Physiology, University of Michigan, Ann Arbor, USA; 3 Department of Physical Medicine and Rehabilitation, University of Michigan, Ann Arbor, USA; 4 Department of Nutritional Sciences, University of Michigan, Ann Arbor, USA

**Keywords:** Bone density, DXA, Weight loss, Obesity, Very low energy diets

## Abstract

**Background:**

The long-term effect of weight reduction on skeletal health is not well understood. The purpose of this study was to examine the impact of an intensive medical weight loss intervention using very low energy diet (VLED) (~ 800 cal/day) that result in significant changes in body weight, on total body bone mineral density (BMD) over 2 years.

**Methods:**

We examined the impact of VLED-induced weight loss on BMD and FFM (Fat-free Mass) after 3–6 months and again while in weight maintenance at 2 years in 49 subjects. The effects of absolute and relative rate of weight reduction assessed by change in weight in kilograms were assessed using general linear modeling, with baseline BMD (or FFM) as a covariate, and age, sex and changes in body weight as primary model predictors.

**Results:**

At the end of 2 years, the average weight loss was greater for men (weight: 23.51 ± 12.5 kg) than women (weight: 16.8 ± 19.2 kg) and BMD loss was greater among women (0.03 ± 0.04 g/cm^2^ vs 0.01 ± 0.04 g/cm^2^) (all *p* < 0.05). After adjusting for baseline BMD, age, and sex, there was a small but significant association between total weight loss and 2-year BMD (β = − 0.001 g/cm^2^; *p* = 0.01). Similarly, there was a significant independent association between total weight loss and 2-year FFM (β = − 116.5 g; *p* < 0.01).

**Conclusions:**

Despite significant weight loss with VLED, there was only a small loss is BMD.

## Background

Among Americans aged 20 years and older, the prevalence of obesity is approximately 35%. Obesity contributes to morbidity and mortality [1]. By 2030, the total healthcare costs attributed to obesity may reach as much as $957 billion [2]. Despite addressing many of the associated co-morbid conditions of obesity, little attention has been paid to the potentially long-term effects of weight change on bone health. Although bone loss is known to occur following bariatric surgeries, little is known about the effects of diet-induced weight loss, and specifically weight loss achieved by a very-low energy diets (VLED).

Bone strength adapts to meet demands of musculoskeletal loading, and therefore, body weight is one of the strongest predictors of bone mineral density (BMD) [3]. Mechanical stimulation of bone leads to osteoblast proliferation, and conversely, reduction in mechanical loading can cause an increase in bone turnover [4]. Lean body mass and fat mass are both independent positive determinants of bone mass, and although BMD may be higher among obese individuals, BMD per unit of BMI is lower and not necessarily protective from the risk of fractures [5, 6]. Excess weight due to adiposity is detrimental to bone, and therefore, obesity itself may predict fractures [7]. Clinical opinions and research pertaining to the impact of weight loss on the bone has been inconclusive. Short-term, longitudinal studies have shown that weight loss achieved through energy restriction can result in an increase in bone turnover that is sustained even during weight maintenance [8, 9]. In addition to BMD, bone turnover markers are thought to predict fracture risk [10]. The long-term effects of VLED on BMD and bone turnover markers following weight loss are yet to be identified.

Very-low energy diets are those that contain ≤800 cal per day, and provide essential daily nutritional requirements. VLEDs have become increasing popular in medically-supervised weight reduction programs for short durations (e.g., 8–12 weeks), with typical weight losses of 1.5–2.5 kg/week, or 18–20% after 8–12 weeks [11, 12]. When incorporated as part of an intensive, behavioral lifestyle program, the majority of VLED induced weight loss can be sustained long-term [11]. This approach offers a less costly, less invasive and practical alternative to endoluminal and surgical approaches.

Due to the growing obesity prevalence, dietary strategies that restrict calories have shown utility for improving metabolic health and health-related quality-of-life [13]. However, the long-term effect of weight reduction on skeletal health is not well understood. The primary objective of our study was to determine the longitudinal association between VLED-induced weight loss on whole body BMD. In addition, we examined whether weight loss influenced an important bone turnover marker.

## Methods

### Design

We included adults who were enrolled in and completed a 2-year weight management program. All participants provided written informed consent and the study protocol was reviewed and approved by the University of Michigan Institutional Review Board. The trial is registered at Clinicaltrials.gov (NCT02043457). The University of Michigan Weight Management Program (MWMP) is a 2-year, multidisciplinary, multicomponent, obesity management program, and has been previously described in detail [13]. Briefly, participants consume a VLED in the form of total meal replacements and are asked to increase physical activity from low to moderate intensity for 40 min per day. Each meal replacement shake contained 160 kcal, 14 g of protein, 23 g of carbohydrates and were fortified with calcium, multiple vitamins including vitamin D. In addition, all participants were prescribed a multivitamin and Vitamin D3 2000 IU daily. After 3 to 6 months, participants were transitioned to regular foods and asked to increase physical activity to moderate-vigorous for 40–60 min per day. Thereafter, the intervention was focused on behavioral and pharmacologic strategies to prevent weight regain. An individualized diet plan comprised of approximately 1200–1800 kcal/day was designed, implemented and titrated to maintain the individual’s reduced weight. A registered dietitian evaluated participants weekly for one month and then monthly for the remainder of the two years. The physician saw participants monthly for three months and then every three months for the rest of the two years. Participants underwent whole body DXA scanning on a (GE Lunar prodigy Advance; Control serial number 070401531487; control model number 7635) by a trained research technician at baseline, after transition to weight maintenance at approximately 6 months, and at the end of the 2-year program. In addition, serum for bone turnover markers was collected to assess the impact of weight loss on bone resorption.

### Study participants

The program was designed for obese (BMI ≥30 kg/m^2^ [or ≥27 kg/m^2^ in Asian Americans]) adults ≥18 years of age. Participants in the program were representative of the demographic of southeastern Michigan, primarily Non-Hispanic whites, educated and employed. The clinic was founded on the principles of the National Heart Lung and Blood Institute (NHLBI) report that addressed long-term weight management for obese individuals (available at https://www.ncbi.nlm.nih.gov/pubmed/24961824) [14]. Participants were excluded if they had unstable psychiatric disorders requiring frequent changes in medications, cancer within the last 5 years (other than non-melanomatous skin cancers), active gallbladder or chronic kidney disease with eGFR ≤35 ml/min, and/or prior bariatric surgery.

Demographic, clinical and metabolic data were collected and entered into a database. This data included participant’s anthropometric, co-morbid health conditions, medications, laboratory, biopsychosocial and imaging data that was updated after each visit.

### Primary outcomes

Morphological Assessment: Whole body BMD, total adiposity assessed as total fat mass (FM) and percent body fat (%BF) were measured by dual-energy X-ray absorptiometry in the Michigan Clinical Research Unit. Determination of FFM was performed for the whole body as a part of the DXA scan. DXA allows for precise assessment of FFM, comprised of fat-free soft tissue and BMD.

Biochemical analyses: Serum cross-linked C-telopeptide (CTX) was collected at baseline, after weight loss between 3 and 6 months, and at the end of the 2-year program for *n* = 25 subjects (selected based on available fasting samples). All blood samples were collected into (2 ml tubes containing EDTA following a 12 h overnight fast to reduce variation associated with circadian rhythms and feeding [15]. The tests were analyzed at the University of Michigan, Michigan Diabetes Research Center (MDRC) Chemistry laboratory. Serum CTX levels were measured by enzyme-linked immunosorbent assay (ELISA). CTX assessments were done in duplicate and all assays were performed in a single run to eliminate interassay variability. The detection limits of this assay were 25–800 ng/ml, and inter and intraassay precision (Coefficients of variation) for CTX assays were < 15%).

### Statistical analysis

Descriptive statistics were used to explore the distribution, central tendency, and variation of each measurement, with an emphasis on graphical methods such as histograms, scatterplots, and boxplots. Descriptive statistics for all demographic and morphologic characteristics were reported at baseline, after short-term weight loss, and at the 2-year time point, as change from baseline. Multiple linear regression analyses with post-intervention outcomes as the dependent variable were used to assess the role of weight loss on change in total body BMD and FFM. All models included baseline morphologic characteristics for muscle, BMD and FFM. For the primary aim, age and weight loss were included as continuous predictors of 2-year BMD and FFM. Several interaction terms were tested to determine the mediating effect of age and sex, on changes in muscle, BMD and FFM. Regression assumptions were checked and appropriate transformations (e.g., log) performed if necessary.

For the sub-analysis, we used a multiple linear regression approach to evaluate the association between changes in body weight, BMD, FFM and changes in serum CTX. Due to the smaller sample of adults with complete morphological and serum data (*n* = 25), models included only age as a covariate, and a change score for body weight, BMD, FFM as predictors of change in serum CTX.

## Results

Forty-nine participants completed both the induction and maintenance phase and had complete DXA scans available at baseline, at 3–6 months and at the end of 2 years. At the end of two years, all participants had significant changes in body weight, mean (− 20.8 ± 11.2 kg), %FM (− 6.7 ± 6.0%), FFM (− 3.45 ± 3.41 kg,), and BMD (− 0.02 ± 0.04 g/cm^2^) (all *p* < 0.05) Descriptive characteristics of participants stratified by sex are presented in Table 1. The decline in BMD was similar for both men and women; however, a statistically significant loss occurred in BMD among women only.

**Table 1:** Morphological characteristics for men and women at baseline and at 2-years

	Men (*n = 29*)			Women (*n = 20*)		
	Baseline	Year 2	Mean Δ	Baseline	Year 2	Mean Δ
Age (years)	51.59 (6.91)	53.58 (6.89)	–	49.30 (7.69)	51.28 (7.70)	–
Body Weight (kg)	123.68 (13.58)	102.56 (15.16)	23.51 (12.45)^†‡^	104.47 (16.78)^*^	89.45 (14.10)	16.80 (7.60)^†^
Body Mass Index (kg∙m^− 2^)	38.58 (3.86)	31.94 (3.92)	7.39 (3.99)^†^	39.17 (4.62)	33.53 (3.92)	6.32 (2.74)^†^
Body Fat (%)	41.79 (4.41)	32.95 (7.22)	8.84 (6.53)^†‡^	51.45 (4.39)^*^	47.88 (5.59)	3.57 (3.29)^†^
Fat Free Mass (kg)	72.72 (6.95)	69.45 (7.55)	3.27 (4.01)^†^	51.26 (5.72)^*^	47.54 (6.07)	3.72 (2.38)^†^
Bone Mineral Density (g/cm^2^)	1.37 (0.06)	1.36 (0.09)	0.01 (0.04)	1.27 (0.07)^*^	1.24 (0.08)	0.03 (0.04)^†‡^
T-score	1.88 (0.77)	1.72 (1.07)	0.16 (0.56)	1.79 (0.84)	1.47 (0.99)	0.32 (0.53)^†^

^*^Significant difference between men and women at baseline (*p* < 0.05)

^†^Significant difference within sex from baseline to 2 years (*p* < 0.05)

^‡^Significant difference between men and women for absolute changes from baseline to 2 years (*p <* 0.05)

**Fig. 1:**
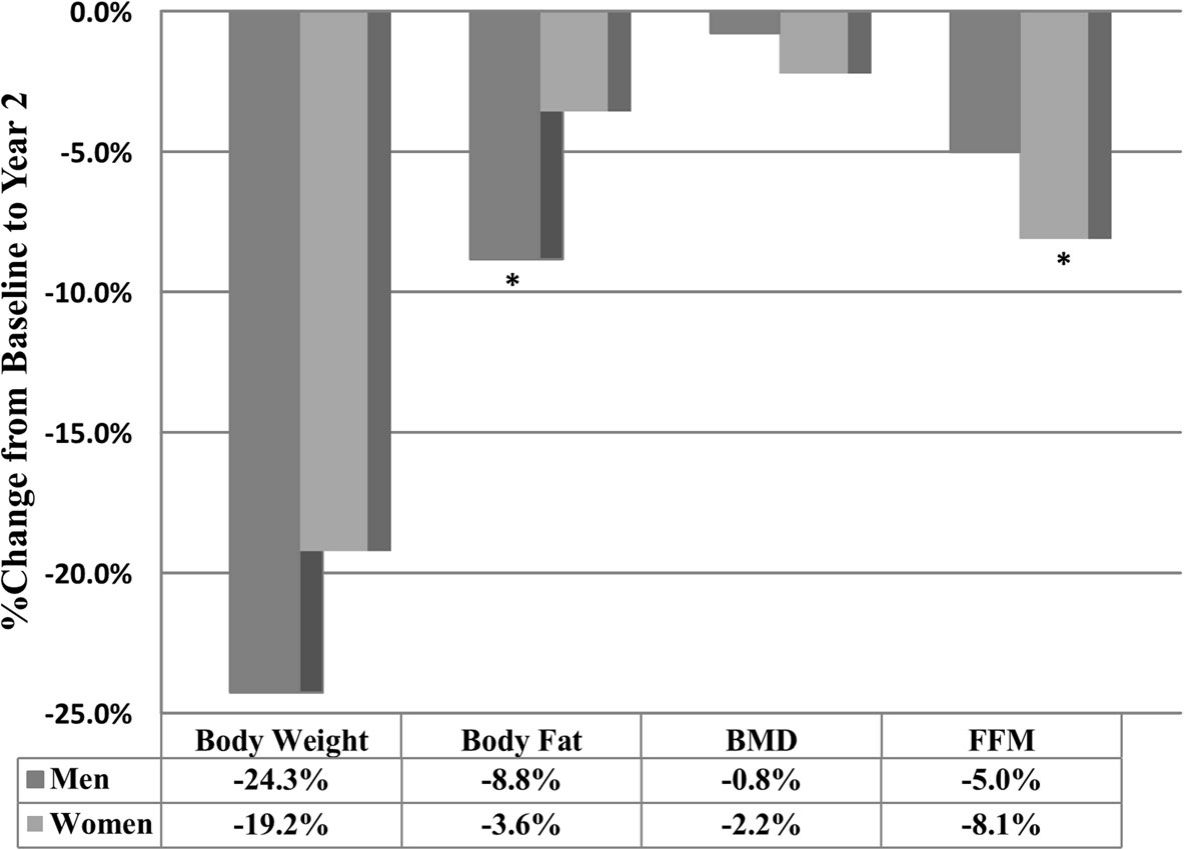
Relative change in body weight, body fat, BMD and fat free mass from baseline to 2 years in men and women

**Fig. 2:**
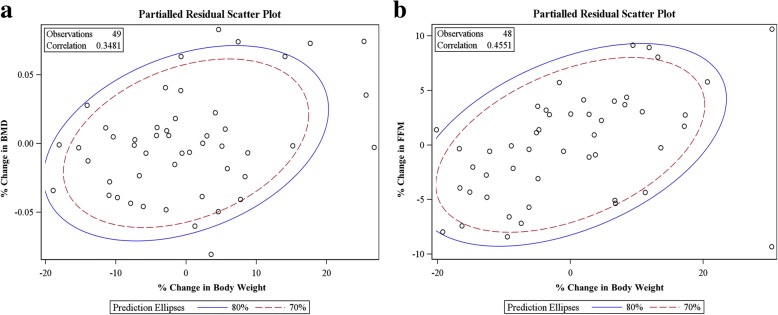
Partial residual scatter plot revealing the correlations between relative changes in body weight and relative changes in BMD (**a**) and FFM (**b**), controlling for age and sex

At two years, the mean weight reduction was greater for men than women; however, change in absolute BMD was greater among women (*p <* 0.05). In multivariable analyses shown in Table 2, after adjusting for age, sex, and baseline BMD, we found that the total weight lost at the end of 2 years was associated with a small but statistically significant loss in BMD at 2-years. There was a 0.001 g/cm^2^ decrease in 2-year BMD per kilogram weight loss. While men lost more FM, women lost significantly more %FFM than men. Figure 1 shows the changes in % body weight, body fat, BMD and FFM from baseline to 2 years in men and women. In addition, there was a significant independent association between total weight lost and 2-year FFM (β = − 116.5 g; *p* < 0.01). Figure 2 includes partial residual scatter plot revealing the correlations between relative changes in body weight and relative changes in BMD (a) and FFM (b), controlling for age and sex.

**Table 2:** Multiple regression showing the associations between changes in body weight (primary predictor) and BMD and FFM at 2-years (dependent variable), after adjustment for age, sex, and baseline values (ANCOVA)

	Model Predictor(s)	β	SE	t	Pr > │t│	Adjusted R^2^
BMD at 2 Years						0.88
	Intercept	−0.084	0.120	−0.680	0.500	
	Age	−0.002	0.001	−2.980	0.005	
	Sex	−0.015	0.014	−1.080	0.285	
	Baseline BMD	1.155	0.082	14.010	< 0.001	
	Change in Body Weight	−0.001	0.001	−2.460	0.017	
FFM at 2 Years						0.94
	Intercept	5.790	6.780	0.860	0.397	
	Age	−0.052	0.066	− 0.790	0.435	
	Sex	−2.420	1.880	−1.290	0.205	
	Baseline FFM	0.950	0.074	12.770	< 0.001	
	Change in Body Weight	−0.117	0.045	−2.620	0.012

In addition, we evaluated the changes in BMD at the end of the intensive phase of weight loss (i.e. after 15% weight loss). There was a small but statistically significant loss in BMD among women only (0.02 ± 0.01 g/cm^2^; *p* < 0.001). In both men and women, the loss in BMD at the end of the intensive phase was significantly correlated to bone loss at 2 years (*r* = 0.77; *p* < 0.01) and (*r* = 0.33, *p* < 0.05) respectively.

In the sub-analyses we evaluated changes in the bone resorption marker, serum CTX and its independent association with changes in body weight, BMD, FFM and %BF. After adjusting for age, change in %BF was the only significant predictor of changes in serum CTX (β = 23.56 pg/mL; *p* = 0.04).

## Discussion

In our study, participants were involved in a two-year weight management program that included a three-month active weight loss phase utilizing an 800-kcal/day standard VLED. Our results show that VLED-induced weight loss can result in a small reduction in total body BMD and FFM at 2-years. Specifically, we found that for every kilogram of weight loss there was a 0.001 g/cm^2^ reduction in BMD and 0.1 kg decrease in FFM at 2-years, after adjusting for age, sex, and baseline BMD. However, it is important to note that this loss of BMD and FFM may also arise from natural consequences of the aging process, as previously described in numerous studies [16]. Indeed, BMD declined substantially in the late peri-menopause, with an average loss of 0.018–0.010 g/cm^2^/yr. from the spine and hip (*P* < 0.001 for both). In post menopause, rates of loss from the spine and hip were 0.022 and 0.013 g/cm^2^/yr. (*P <* 0.001) [17]. Serum CTX levels increased following the intensive phase and remained stable in the maintenance phase. Neither baseline serum CTX nor changes in CTX levels following weight loss were associated with bone loss.

The effect of weight loss on the bone has been controversial with inconsistent clinical opinions and research findings. In the Dubbo Osteoporosis Epidemiologic study, weight change was found to be an independent predictor of rate of bone loss [18]. Moderate weight reduction (greater than 5%) can negatively influence BMD especially if achieved purely through calorie restriction [19, 20]. This was also observed in the LOOK AHEAD study, and while intensive lifestyle interventions resulted in better glycemic control and weight loss, a statistically significant loss in BMD was noted at the total hip and femoral neck [21]. The impact of calorie restriction on BMD is seen in younger individuals as well as premenopausal women therefore suggesting that the mechanism is not solely related to age and the effects of sex steroids [22, 23]. In middle-aged individuals, weight variability has been shown to increase the risk of hip fractures [24]. However, the long-term impact of calorie restriction on fractures is not known [23, 25]. In recently published data from the LOOK AHEAD study, long term weight loss in overweight and obese adults with type 2 diabetes mellitus was not associated with an increase in overall risk of fractures but maybe associated with an increased risk of frailty fractures [26]. A few other studies evaluating the long-term effects of > 5% weight reduction did not use severe calorie restricted diets [18, 27, 28]. Due to significant energy restriction, VLEDs induce greater weight loss than moderate calorie restricted diets. Citing the need for further research, a meta-analysis performed by Zibellini et al.*,* found that low calorie or VLED’s did not cause a reduction in BMD; however, the majority of studies evaluating the impact of VLEDs on bone loss have been limited to one year [25]. In addition, low energy diets that are supplemented with calcium or high proteins are known to mitigate the rise in bone turnover [29]. In our study, although the changes were statistically significant, the change in BMD was small, and thus may not necessarily be clinically relevant in the short term, or with respect to future fracture risk. We postulate that the loss in BMD was likely attenuated by the macro/micro-nutrient composition of the meal replacement and adequate vitamin D and mineral intake and less sedentary behavior.

Bariatric surgery remains the most effective form of treatment for severe obesity and historically, Roux-en-Y gastric bypass (RYGB) procedure was the most commonly performed procedure. Though these surgeries are very effective, the risk of bone loss associated with gastric bypass surgery is well documented [30–33]. Surgical procedures are associated with decreases in bone mass and increases in bone turnover markers [34]. Prospective studies have shown that bone loss following gastric bypass preferentially affects the hip [32, 35]. Of the various bariatric procedures, sleeve gastrectomy is likely associated with less bone loss than RYGB although the studies are small and more data are needed [36]. We postulate that the derangements in calcium and Vitamin D absorption are likely to play a greater role in bone loss after surgical procedures. VLEDs may therefore represent a safer, non-invasive alternative to weight loss without the negative impact on bone.

Evidence pertaining to the long-term impact of weight loss on bone turnover is lacking. Some studies have shown low bone turnover in obesity while others have contrary findings [37, 38]. Following bariatric surgery, Balsa et al. showed an increase in bone turnover markers [39]. In our study, we did not find a correlation between serum CTX and BMD.

## Limitations

In this study, DXA imaging at one year was not available. With this information, we would have been able to assess the sequential changes in BMD in individuals whose weight remained stable and those who experienced weight cycling. In addition, we looked at the total body BMD rather than routinely used sites to assess fragility such as the lumbar spine, femoral neck or hip, and this may result in under diagnosis of osteoporosis [40]. Although the utility of DXA in obese individuals is debatable, DXA remains the gold standard and the only available test for measuring BMD in clinical practice [41]. In addition, whether changes in total body water with these diets affects fat and fat-free mass assessments via DXA remains unclear. Our sample size for subgroup analyses on bone markers was small, and thus we had to combine men and women. Future larger studies should aim to determine the longitudinal, dimorphic patterns of weight loss and BMD changes, taking into consideration the mediating influence of serum BTM changes. Lastly, although moderate intensity and regular physical activity was prescribed as part of the program, physical activity and participation was self-reported. Since physical activity and exercise are linked with bone health, future efforts are certainly needed to determine if objectively measured exercise during VLED interventions can ameliorate changes in BMD and FFM. Our future studies will prospectively evaluate key variable such as gonadal status, use of bone altering medications and physical activity.

## Conclusion

Obesity has negative effects on bone metabolism and is associated with a number of cardio-metabolic conditions that pose threats to bone health. We have shown absolute weight loss that can impact BMD. Although VLED can promote significant weight loss the decline in BMD is minor with unclear clinical applicability and must be weighed against the myriad of other benefits resulting from weight loss.

## Abbreviations

BMD: Bone Mineral Density
BMI: Body Mass Index
CTX: Serum cross-linked C-Telopeptide
DXA: Dual X-Ray Absorptiometry
FFM: Fat-Free Mass
FM: Fat Mass
VLED: Very Low Energy Diets
